# Monitoring Fatigue Status with HRV Measures in Elite Athletes: An Avenue Beyond RMSSD?

**DOI:** 10.3389/fphys.2015.00343

**Published:** 2015-11-19

**Authors:** Laurent Schmitt, Jacques Regnard, Grégoire P. Millet

**Affiliations:** ^1^Centre National de Ski Nordique et de Moyenne Montagne, Ecole Nationale des Sports de MontagnePrémanon, France; ^2^Faculty of Biology and Medicine, Institute of Sport Sciences, University of LausanneLausanne, Switzerland; ^3^Unité de Recherche EA3920, Marqueurs Pronostiques et Facteurs de Régulations des Pathologies Cardiaques et Vasculaires, Hôpital Universitaire de Besançon, Université de Franche-ComtéBesançon, France

**Keywords:** heart rate variability, RMSSD, overreaching, fatigue, monitoring, physiologic

## Abstract

Among the tools proposed to assess the athlete's “fatigue,” the analysis of heart rate variability (HRV) provides an indirect evaluation of the settings of autonomic control of heart activity. HRV analysis is performed through assessment of time-domain indices, the square root of the mean of the sum of the squares of differences between adjacent normal R-R intervals (RMSSD) measured during short (5 min) recordings in supine position upon awakening in the morning and particularly the logarithm of RMSSD (LnRMSSD) has been proposed as the most useful resting HRV indicator. However, if RMSSD can help the practitioner to identify a global “fatigue” level, it does not allow discriminating different types of fatigue. Recent results using spectral HRV analysis highlighted firstly that HRV profiles assessed in supine and standing positions are independent and complementary; and secondly that using these postural profiles allows the clustering of distinct sub-categories of “fatigue.” Since, cardiovascular control settings are different in standing and lying posture, using the HRV figures of both postures to cluster fatigue state embeds information on the dynamics of control responses. Such, HRV spectral analysis appears more sensitive and enlightening than time-domain HRV indices. The wealthier information provided by this spectral analysis should improve the monitoring of the adaptive training-recovery process in athletes.

The optimization of the training process in elite athletes requires the quantification of the training loads (Borresen and Lambert, 2009) and a thorough analysis of the training program (Tønnessen et al., 2014). Adjusting the training content aims at allowing optimal improvement in fitness. Therefore training has to be customized according to environmental conditions (e.g., altitude Schmitt et al., 2008; ambient temperature Brocherie et al., 2014), cross-transfer between training components (Millet et al., 2002), training phases (Issurin, 2010). It is also crucial to take into account any athlete's fatigue state and/or performance responses to the training load. Among the methods available for diagnosing a particular kind of “fatigue” (e.g., non-functional overreaching NFOR or overtraining, Meeusen et al., 2013), the heart rate variability (HRV) is widely used as its alterations depend largely on changes in cardiac autonomic control which continuously attempts to adapting cardiovascular function. HRV has been assessed either at rest, awake (Schmitt et al., 2006; Plews et al., 2013a), or sleeping (Pichot et al., 2000; Garet et al., 2004), during exercise (Sandercock and Brodie, 2006) or during the post-exercise recovery phase (Buchheit et al., 2007; Seiler et al., 2007; Hug et al., 2014).

In a recent review (Buchheit, 2014), the pros and cons of these different measures have been elegantly presented. The suggested outcome was that the most useful resting HRV indicator would be the time domain index RMSSD (square root of the mean of the sum of the squares of differences between adjacent normal R-R intervals) measured during short (5 min) recordings in supine position upon awakening in the morning. The method gathers several advantages as an easy and quick accessibility, a short recording time not disturbing the athlete's recovery and a lower sensitivity to breathing pattern than spectral variables (Saboul et al., 2013). For all these reasons, the logarithm of RMSSD (LnRMSSD) is described as the “*most reliable and practically applicable measure for day-to-day monitoring*” (Plews et al., 2013b) and different recommendations have been proposed to improve the quality of the “fatigue” diagnosis: the use of weekly average (Plews et al., 2013a) of a minimum of three (ideally, randomly selected) measures of Ln RMSSD per week (Plews et al., 2014); a 7-day running average of LnRMSSD (Plews et al., 2012) instead of daily measures; the use of LnRMSSD/RR ratio (Plews et al., 2012, 2013b; Buchheit, 2014) for identifying any vagal-related saturation phenomena; the interpretation that coefficient of variation (CV) of LnRMSSD is linearly decreased toward NFOR (Plews et al., 2013a; Buchheit, 2014). All these information are useful and relevant, increase the signal-to-noise ratio, the reproducibility of these measures and therefore improve the quality/robustness of the monitoring of the “fatigue” status.

However, despite its accessibility/simplicity, even with all the above-mentioned methodological improvements (see Plews et al., 2013a; Buchheit, 2014, for further details), in our view, using only time-domain HRV indices for monitoring the training status in elite athletes has limitations, and might even lead to a dead-end (Schmitt et al., 2013, 2015). RMSSD and its proposed derivatives are taken as a vagal index (Berntson et al., 2005). Indeed, an increase in vagal heart control reflects often a fitness improvement, while athete's fatigue and performance impairment are often concomitant with a decreased vagal HRV (Pichot et al., 2000; Iellamo et al., 2002; Gratze et al., 2005). However, vagal heart control is influenced by sympathetic activity that can either impede or bolster it (Task-Force, 1996). In addition, the interplay between sympathetic and parasympathetic influences changes along the resting—exercising scale, to adapt the distribution of muscle blood flow and heart work (Harms et al., 1998). Thus, autonomic patterns differ according to different functional requirements in different training phases and in different types of sport activity. As from lying supine to standing (Stewart, 2012), from rest to various exercising levels the fine tuning of autonomic adjustments relies on fine resetting of baroreflex activity (Ogoh et al., 2007) with a complex interplay, as e.g., modulation of carotid-aortic baroreflex activity by low pressure cardiopulmonary receptors (Halliwill et al., 2014). Indeed, performing at high level requires an optimal interplay of parasympathetic and sympathetic controls (Hedelin et al., 2001; Pagani and Lucini, 2009; Hug et al., 2014). RMSSD does not provide any information on the sympathetic-related modulation. A decrease in RMSSD may have also a biased interpretation due to vagal saturation (Kiviniemi et al., 2004), as it could result of a sympathetic overactivity combined with a vagal-related saturation. By measuring RMSSD alone in the sole supine position, the use of LnRMSSD/RR ratio is not unambiguous since an increase in this ratio may be taken in both opposite ways (either reflecting a fitness improvement or an increased fatigue). Lastly, the values obtained from the sole lying data recording do not provide any clue about the preserved or altered ability to dynamic control adjustment.

In our view, recording HRV clues in both supine and standing positions is also convenient and provides more information about the actual autonomic settings, their interplay and how they are resorted (Schmitt et al., 2013, 2015). We believe that the analysis of changes in HRV between supine and standing provides information about the ability of autonomic control to assume resetting for functional adaptation. In orthostatic tolerance assessment, HRV patterns in both supine and standing positions are affected by different involvement of cardiopulmonary receptors, i.e., cardiac preload and hence tuned changes in plasma volume and/or peripheral vasomotor tone (Iwasaki et al., 2000; Stewart, 2012). Among other factors, these latter parameters likely support changes in autonomic patterns and HRV also during exercising settings, upon different training loads and phases, as in day to day changes in sleep quality or appetite.

A recently published study accurately displayed how individual patterns of spectral analysis of HRV divert in “fatigue” states from “no fatigue” condition (Schmitt et al., 2013), and the data analysis describes the clustering of different types of fatigue through mathematical proximity of heart rate and main variables of spectral analysis (Schmitt et al., 2015). These distinct patterns encompass increases and/or decreases in HR as well as in spectral low frequency (LF) and high frequency (HF) components, and these changes are differently sized in supine and standing positions, and also sometimes contrariwise directed in each position. A main outcome of the analysis was that supine and standing HRV variables were fully independent, and non-commutable in the clustering of alterations from the individual normal “no fatigue” patterns. Indeed, low pressure baroreceptors are not activated similarly in supine and standing positions, and other inputs of autonomic control are likely differently active in each position. This study highlights the importance to combine HRV analysis in supine and standing positions. The new HRV-based sub-categories of “fatigue” may open doors for a more precise monitoring of athlete status and for different specific recovery strategies (that remain to be validated). We believe it represents an interesting step forward in using HRV for diagnosing NFOR and overtraining in athletes.

In summary, RMSSD measures and their derived variables have an effective practical usefulness, which can help the practitioner to identify a global “fatigue” level. However these variables do not allow the clustering of different sub-categories of “fatigue,” at variance with the spectral HRV analysis in both supine and standing positions, which likely consider the current ability to control in a dynamic setting.

## Conflict of interest statement

The authors declare that the research was conducted in the absence of any commercial or financial relationships that could be construed as a potential conflict of interest.
